# The introduction of microcomputers into the undergraduate teaching laboratory

**DOI:** 10.1155/S1463924686000020

**Published:** 1986

**Authors:** H. Elliott, G. N. Elson, D. Allerton

**Affiliations:** Science Department Stockport College of Technology Wellington Road South Stockport SK1s 3UQ UK


Journal of Automatic Chemistry/*Journal of Clinical Laboratory Automation*, Volume 8, Number 1 (January–March 1986), pages 6–11

### The introduction of microcomputers into the undergraduate teaching laboratory

**H. Elliott, G. N. Elson and D. Allerton**

*Science Department, Stockport College of Technology, Wellington Road South, Stockport SK1 3UQ, UK*

### Introduction

The role of the technician in the chemical laboratory is undergoing a dramatic change as the computer makes inexorable inroads into the field of laboratory operations and instrumentation. The use of the computer in laboratories has progressed from the single mainframe in the large research institutions of 20 years ago, through the free-standing micros in the mid 1970s, to the computers dedicated to individual instruments widely used today. These developments have resulted in an increasing range of laboratory employees, from junior technicians to section leaders, routinely coming into contact with computers. The education sector has not, in general, responded to these changes by appropriate changes to curricula to maintain the relevance of the education and training provided for potential laboratory employees. This paper describes the developments undertaken in one science department to attempt to satisfy the new demands being made on laboratory staff.

### The department

The Department of Science at Stockport College of Technology runs a range of part-time courses for technicians released for one-day per week from science-based industries. The range of courses includes the National and Higher National Certificates of the Business and Technician Education Council, and the Honours Degree in Chemistry of the Council for National Academic Awards. The students are sponsored by a variety of employers, including a range of small- and medium-sized companies and one major supplier, ICI Pharmaceuticals, which employs approximately one-third of the student body.

The Department's laboratories are adequately equipped for its teaching role with spectrophotometric, chromatographic and electrochemical instrumentation. In common with most other educational institutions, however, the current financial climate does not allow its acquisition of new instrumentation to keep pace with the advances being achieved by the instrument manufacturers. The purchase of a new item of equipment must be justified on the basis of its use in teaching rather than research, which prohibits the introduction of automated instrumentation based on dedicated computers. The available instruments have been acquired over a period of 20 years, but, whilst not comparable to the state-of-the-art equipment available to the major employer of students seconded to the Department, are reasonably representative of those used by the majority of employers who have neither the resources, nor the throughput of work, to justify frequent purchase of the most modern automated equipment.

### Analysis of the educational problem

Discussions with employers and students during 1981/1982 suggested that some measure of computer competence was desirable for all laboratory staff, but revealed no consensus on the nature or extent of the expertise required. At that time, many laboratories were buying microcomputers before identifying specific applications and often without having any clear conception of their potential uses. Some laboratories had purchased several non-compatible systems, whilst others had given no consideration to the potential applications of microcomputers to their operations. Given the lack of clear guidance on future requirements, the Department formulated three general aims.

To provide students with:

(1) An awareness of the applications of microcomputers in the laboratory.

(2) Sufficient experience to use laboratory microcomputer systems with confidence.

(3) The ability to judge whether a specific problem is suitable for solution using computer techniques.

It was considered that these aims could be achieved through exposing the students to the use of microcomputers in the following areas:

(*a*) Data handling.
(*b*) Data acquisition.
(*c*) Instrument control.

Whilst it seemed unlikely that any student would be required to write complete software packages as part of his job without further extensive training, some experience of programming techniques seemed necessary to achieve maximum benefit from computer-linked experiments in the teaching laboratory. The entire programme of tuition in computing was to be integrated into the chemical curricula so that students could develop the concept of the computer as a major resource in the practice of chemistry, rather than as an optional extra.

The Department's task was, therefore, identified as developing the facility to provide its students with experience of:

(i) The use of free-standing microcomputers for handling and presentation of data relevant to their chemical studies,

(ii) The use of microcomputers interfaced to a range of analogue and digital instrumentation for data acquisition and instrument control.

These developments were to be undertaken within the constraints of limited financial and experienced human resources. The Department's collective mind was further concentrated on this task by the Council for National Academic Awards (CNAA) imposing, as a condition of reapproval of the BSc (Honours) Chemistry Course, the requirement that a report on progress in the field of laboratory computing be submitted by September 1984.

### Hardware

When this work was commenced in early 1983, the BBC Model B microcomputer was finding widespread applications in UK educational institutions. The Department already possessed a BBC system, including a disk drive and printer; and its built-in interfacing facility, high-resolution graphics and ease of programming in assembly language seemed to make it ideal for the above purposes. This great potential was, however, unlikely to be realizable in the 18 months' timescale set for this work since little validated software was available.

The Department's requirement was for a proven combination of hardware and software on which the staff could rapidly gain experience and produce student experiments in the minimum time. A survey of commercial software indicated that the most appropriate system was the Apple IIe. The system purchased was the Apple IIe with 64K memory and 80-column card, dual $5\frac{1}{4}$ in disk drives, monitor and Apple dot matrix printer controlled by a Grappler interface card.

The Department's interfacing requirements were for a system that would allow communication with a range of analogue and digital instrumentation. The range of experience required by the students demanded that the limited number of computers available be rapidly transferrable between several instruments. Two interfaces were purchased to supply this versatility, the Adalab (Interactive Microware, Inc.) and the Super Rexagan (Imperial Chemical Industries).

### Free-standing data handling

The principal constraint on student laboratory work in advanced chemistry courses is the time required to calculate results. The aim of this part of the work was to provide data processing techniques which would enable students to carry out more sophisticated analyses on existing experiments, to introduce experiments which generate large amounts of data and to establish the analysis of errors as a standard technique. All students attend a series of computing courses which includes programming in BASIC and the development of programs involving the standard numerical methods required for the calculation of results of specific experiments from the laboratory schedule. The course involved the use of the College PDP II minicomputer.

The experience so gained provided the student with the confidence to use the computer in data handling and also enabled a library of suitable programs to be compiled. This library was enhanced by published general-purpose BASIC programs [1 and 2]. The programs provided were, however, usually each restricted to one specific experiment. The provision of a comprehensive data-handling facility, including graphics routines, within the short time available required the purchase of fully developed commercial software packages; at that time suitable packages for the BBC and PDP II were not available, but a variety of software for the Apple IIe was on the market.

The choice made was a series of compatible programs by Interactive Microware, Inc. comprising 'curve fitter', 'scientific plotter' and 'vidichart'. An attractive feature of these programs was that they are listable for modification in BASIC. Initial trials showed that the package contained all the required facilities, but that the procedures for transferring data between the three programs required considerable experience. The manuals were comprehensive, but incomprehensible without considerable study; an early decision not to subject the students to the manuals was taken.

The unmodified programs were not immediately suitable for student use since the time required to gain familiarity was not available. Those students who did attempt to use the supplied programs suffered considerable frustration and wastage of time, which often led to disillusionment with laboratory computing. The most efficient use of student time required that for each experiment a disk file, which would enable the data produced to be analysed in a sequential process under the control of the program, be developed. The development work undertaken by the staff has, therefore, involved the production of EXEC files which control the operation of the appropriate sections of the programs in the correct order. This task has proved extremely time-consuming but several experiments have now been formatted in this manner. Student response to the developed programs has been favourable; the success of these packages has made a great contribution to the Departmental aim of teaching the student when, as well as how, to use the computer for data analysis.

A considerable development was achieved by linking the Apple IIe and PDP II, using a California Computer Systems Model 7710 Asynchronous Serial Interface Board and under the control of the ASCII Express (South Western Data) software package. This enabled the commercial software to be readily combined with the students' own programs stored on the PDP II. A typical experiment involves a series of potentiometric titrations. The data are entered at the Apple IIe keyboard and titration curves produced by 'scientific plotter'. A fitted curve is produced by 'curve fitter' and the fitted data sent by ASCII Express to PDP II for end-point determination and error estimation using student programs. The student is provided with a hard copy of the original data, the titration curves and a print-out of the end-points with full error analysis.

### Computer-interfaced instrumentation

The aim in this part of the work was to simulate the facilities provided by dedicated-computer instrumentation, using the machines already in the Department enhanced by possible future purchases. The majority of existing instruments involved electro-mechanical controls and provided analogue signals. Only the most recently purchased instrument provided a digital signal and the facility for electronic control. The task was to use the Adalab or Super Rexagan interface systems to acquire data from a range of instrumentation for processing, either by available commercial software or by packages produced in-house.

### *The Adalab system*

*Use in analogue data transfer:* the Adalab board is designed to fit into one of the slots in the Apple IIe. It contains facilities for input and output of both analogue and digital signals and is supplied with the 'Quick I/O' software package to control transfer of information. Connections to analogue instruments were made through the Ada-Amp output amplifier. Whilst data acquisition using this system is, in principle, straightforward, considerable initial difficulties were encountered in producing satisfactory results. The problem was that the output from the instruments used invariably needed some form of processing before entering Adalab. It was necessary to analyse the signal characteristics and convert it to a form suitable for Adalab, usually by filtering out noise or by using a backing-off device to overcome zero offsets. Solution of these problems required a detailed knowledge of analogue circuits and proved more time-consuming than should have been necessary because of their superficial treatment in the manuals. The Adalab 'Quick I/O' package has proved a reliable system of acquisition of analogue signals from a range of instruments, once the initial problems of familiarity were overcome. The software package 'Lab Data Manager 1' (Interactive Microwave, Inc.), incorporating 'vidichart', 'vidisampler', 'vidimemory' and 'stripcharter', was chosen to process and display data because it is compatible with Adalab. An initial irritation with this package was that, although advertised as a single program, it is supplied as its individual constituents requiring configuration into the complete package. More importantly, problems in printing data held up the full commission of the package for several months. The fault was eventually traced to an incompatibility of the 'Stripcharter' program with the Apple dot matrix printer, which was unexpected given that the hardware and software were purchased together as a complete package.

The LDM1 package is designed to operate on data contained in four buffers, each of which can accommodate 1024 points. The data in the buffers may be subjected to a wide range of operations, transferred to and from a 128K extended memory card, displayed on the screen and sent to the printer. The types of applications required for student use were the acquisition of raw data from the instruments, processing to remove unwanted features, comparison with references and the production of improved displays either on a chart recorder or on the printer.

Considerable difficulties were initially encountered due to the small size of the data buffers. It was found, for example, that the average infra-red spectrum in the region 4000–650/cm saturated all four buffers. Transfer of data to and from extended memory was less straightforward than suggested in the manual, and, at the maximum sampling rate of 20 samples/s, the maximum of 4000 data points on the screen did not allow a complete spectrum to be displayed. The technique eventually determined was to record the data in extended memory then call up blocks on which the required operations and printing could be carried out.

Experience showed that the maximum sampling rate of 20/s was rarely needed. The quality of the recorder of a typical low resolution infra-red spectrophotometer could be equalled using $\frac{1}{2}$K of data points from a sampling rate of 2/s; highly satisfactory quality could be obtained from a rate of 10/s. A remaining problem is that of correlating the calibration of data in the computer with the source instrument. This is often a consequence of the age of the instrumentation; non-linear wavelength calibration of infra-red spectrophotometers is a typical example.

The LDM1 package, though effective, requires considerable experience to use with confidence, so is not suitable for direct student use. The requirement was, therefore, to develop EXEC files which students could use for specific experiments. Where this has been achieved, the package has enhanced the techniques available in the Department. Examples are base-line subtraction, peak integration and production of derivative curves. These operations are limited by the size of the data buffers and the requirement to leave one buffer vacant to store the result. For example, if the subtraction of solvent absorption from a spectrum is required, three buffers must be used to hold object, solvent and difference spectra. Since only four buffers are available, each of the three spectra are limited to the 1024 data points which may be accommodated in a buffer.

The display of data has been improved by plotting the final form through LDM1 on an Iwatsu SR-6602 plotter. The resultant improvement in quality of data has led to better experimentation and to an improved student awareness of the constraints which instrumental deficiencies place on the significance of experimental results.

Successful acquisition and processing of analogue data has been achieved using this system in conjunction with the Perkin-Elmer 237 infra-red spectrophotometer and the EDT Model E 100 DP polarograph. A further development has been the acquisition of data from the Perkin-Elmer Sigma 3 gas-liquid chromatograph using the 'Chromatochart' software package (Interactive Microware, Inc.). This package provides full facilities for processing and displaying GLC data, and is sufficiently user-friendly to be operated directly by students without in-house amendment.

*Use in serial data transfer:* the only instrument in the Department capable of digital control at the beginning of this work was the Pye-Unicam SP8-250 ultra-violet/visible spectrophotometer. This instrument was supplied with an RS232C interface, but without software support. The manufacturer has not been able to supply software to operate this instrument, other than details of operating protocols for use in self-developed programs.

Transmission of serial data is too fast for BASIC, so requires data handling at machine-code level. Two-way communication has been achieved through a California Computer Systems Model 7710 asynchronous serial interface board using the machine-code subroutines of the 'Quick I/O' package, which have been incorporated into BASIC programs to provide suitable software for data acquisition and instrumental control.

The major problem experienced in serial data acquisition was the output as ASCII character strings produced by the SP8-250, which is not suitable for use in the available data-processing packages. Conversion can be achieved using the VAL command in BASIC, but the use of the high-level language places a considerable constraint on the speed of data handling. The procedure for data acquisition is to store the data from the SP8-250 in a buffer until processed by the Apple, then to store on disk. The data files are called from disk by the LDMI software package and processed. Whilst this technique provides the full processing of spectral data required, it does not allow display of spectra in real-time on the monitor. It has also been found that significant data-transmission errors occur at high baud rates; the maximum satisfactory rate has been found to be 300 baud.

Control of the operation of this instrument from the Apple has presented major difficulties, due in part to the incompleteness of the information by the manufacturer and the complexities of the operating codes. It has, so far, been found to be possible to set operating parameters on the SP8-250 and transfer to the Apple for disk-storage. The instrument parameters can then be set from these format files through the serial interface – a series of files of operating conditions for specific experiments has been compiled. Communication between the Apple and SP8-250 has not yet been extended to setting or changing instrumental conditions from the keyboard.

### *The Super Rexagan system*

The Super Rexagan was originally designed by ICI as a comprehensive analogue and digital input/output interface for Apple and Pet microcomputers. Shortly after the Department's purchase of the Apple system, critical errors in both hardware and software were detected. ICI had, by then, withdrawn support for the Apple system. Development of the BBC-compatible version was by then underway, but almost two years were lost in the process. A programme of development work on the BBC/Super Rexagan system is now well advanced.

The Super Rexagan consists of a 19 in rack which houses the system's power-supply and a data bus into which function cards are slotted. Communication with the BBC microcomputer is through a personality card connected to the 1 MHz bus. Each function card has selectable address links allowing communication through the &FCCO–&FCFE regions of memory, which are reserved for user applications. The function cards implemented so far are (1) 16-channel 12 bit resolution analogue input (AI), (2) 16-channel digital output (DO), (3) four-channel 12 bit resolution analogue output (A), (4) serial input/output (SIO).

*Use in infra-red spectrophotometry:* the aim of this project was to simulate the data-handling facilities of a commercial data station. The development work was carried out using the Perkin-Elmer 237 spectrophotometer. An immediate problem was the lack of available memory when using the high resolution modes required for spectral display. This was overcome by purchase of the Aries-B20 RAM expansion (Cambridge Computer Consultants), which removes the screen display from the main memory leaving approximately 27K for program and data.

The acquisition of the analog output from the spectrophotometer through AI, real-time display on the monitor and saving of data on disk were achieved using BASIC programs. The requirement for comparison of spectra, and hence of holding two spectra simultaneously in memory, placed a limit of 10K on each spectrum. The use of 5 bytes by each integer variable used to represent the spectral data limited the number of data points to 2000. When recording the full 4000–650/cm spectral range of the spectrophotometer, the sampling interval was approximately 1.5/cm.

Acceptable spectra were produced under these conditions, but higher resolution work required the selection of smaller spectral ranges. The package includes smoothing routines using the moving boxcar technique with variable averaging, ordinate and abscissae scale expansion, and facilities for the overlay, addition and subtraction of spectra.

The Perkin-Elmer 237 gives no signal to indicate wave-number, relying on pre-printed charts for calibration. The linear wavenumber scale used by this instrument was used to calibrate the spectral display on the monitor. The scan and data acquisition are started and stopped simultaneously by switching the scan motor through the digital output card (DO). The wave-number scale on the display is then produced by calculation based on the wave-number range and the number of data points taken.

The derivation of maximum benefit from the improved spectra produced by the data-processing techniques requires the production of spectra as hard copies. Screen dump techniques in the high resolution modes used for spectral display were found to be slow and to produce poor results. This problem was solved by using the analogue output card (AO) linked to a Kipp and Zonan BD9 two-channel recorder. The package has the option to plot any spectrum simultaneously on screen and recorder, one recorder channel being used for the spectrum and the other for the wave-number calibration.

The greatest benefit gained by students from use of this package is an appreciation of the advantages of software techniques. The spectrum of a sample is recorded on the spectrophotometer recorder as the data are logged by the computer. The various operations may be carried out on the data, and when the required quality is obtained, a hard copy obtained for comparison with the original. An unexpected success was the enthusiasm for exploring group-frequencies generated by the comparison facility, drawing on the library of spectra being compiled.

*Use in atomic absorption spectrophotometry:* The Department has recently purchased a Perkin-Elmer 2280 atomic absorption spectrophotometer fitted with an RS232C serial interface. The interface is designed to operate through a teletype, having only output lines implemented. These outputs have been linked to the BBC microcomputer through the Super Rexagan serial input/output card (SIO). The data are transmitted when the read button is pressed in the print mode, and read by an assembly language routine. Considerable difficulties were experienced initially in interpreting the data, which are transmitted as ASCII strings. Although the data were being received, the characters displayed by the BBC did not correspond with those shown on the spectrophotometer's digital display. This problem has not yet been fully resolved, uncorrupted data being received only at 110 baud.

The standard atomic absorption experiment performed by students is to read a series of standard solutions for the metal under test, plot a calibration curve then determine the concentration of the metal in an unknown. The software package being developed reads the data for the standards and plots the standards with automatic scaling. The linearity of the data are tested using least squares techniques and the coefficient of correlation calculated. If this coefficient is outside pre-set limits, the equation of the calibration curve is calculated using a polynomial least squares method. The unknown is read, plotted on the graph and the concentration calculated using the equation of the curve. The student is provided with a print-out including the readings for the standards and unknown, the calibration graph, the equation of the curve, and the unknown concentration, together with an estimation of the error.

### Discussion

The prime requirement of computing facilities in educational laboratories is flexibility of application; few institutions will have the throughput of work or the resources to justify the dedication of a computer to an individual instrument. For teaching purposes, both hardware and software must be reliable and easy to use; the non-enthusiasts in computing among the students can be irrevocably turned against laboratory computing if valuable practical periods are wasted through use of faulty packages.

The experiences of this Department suggest that entirely suitable packages for student use are not commercially available. Our initial response to the apparently well tried and documented software packages purchased was disappointment – their user-friendliness was considerably less than implied. Once familiarity was established, however, the Interactive Microware software did supply most of the required facilities. The major difficulty proved to be that each package contained more facility than the student would normally require and much practical time could be wasted by working through unnecessary sections of program.

The input of staff time required to convert commercial software into routines suitable for student use was many orders of magnitude greater than expected. The most efficient way to accomplish such a task is for one person to devote himself completely to it, which is most unlikely to be possible given the nature of the duties of the lecturer. Frequent interruptions for teaching and other duties break the concentration on the task and cause inordinate extension of the work. The packages written in-house for the BBC microcomputer have proved most successful. Each of these was designed to strictly defined objectives, so is less versatile than the commercial packages. The cost in terms of staff-time has been considerable, which suggests that a market for suitable laboratory software packages exists.

The ideal software for educational use would be composed of modules designed for specific purposes, such as data logging curve fitting, graph plotting and statistical analysis. Each module would be available in both BASIC and machine code versions. The modules would have compatible methods and formats of data input and output and would be easily combined into complete packages for specific experiments. Each module would require the minimum number of simple operations from the student and would be accompanied by precise documentation readily understandable by the non-specialist. Our experience suggests that any enterprising software house which addressed itself to this task would find great demand from education and from small industries.

The timescale set for the Department to implement the techniques of laboratory computing was very short. Despite the many problems described, the aim has been achieved. The staff involved have developed greater competence in computer systems, the techniques of interfacing and software development than would have been the case if immediately useful systems had been commercially available. The techniques assimilated have both enabled later laboratory developments to be made more rapidly and provided the basis for the preparation of lecture courses to accompany the students' practical work.

The student response to the introduction of computer-assisted data handling has been most favourable. The most important attributes are that the use of the computer must be seen as arising naturally from the experimentation undertaken and must provide obvious improvements in the results obtained. Only well-designed experiments of this type can equip the student with the ability to distinguish those applications where the use of the computer is purely cosmetic from those where it provides real benefits. The programme currently provided for the students, involving a combination of experience in chemical programming with the use of prepared laboratory packages, has succeeded in establishing the concept of the computer as an essential tool in the practice of chemistry in the majority of the students. The aversion to numerical techniques commonly displayed by part-time chemistry students has been largely overcome and most now feel comfortable using sophisticated methods of data processing.

The success of the current programme of work in establishing favourable student attitudes to laboratory computing is demonstrated by the suggestions for extension into other areas now being received from the students. The aim of the next phase is to improve the data-handling techniques now in use and to extend them for further applications. The preliminary work in instrumental control must be consolidated, pending the purchase of further electronically operated instruments. Perhaps the greatest challenge to the staff is to develop the available techniques with sufficient rapidity to keep abreast of the increasing experience of computing of students now entering the Department's courses.

### References

1. Lee, J. D. and Lee, T. D., *Statistics and Computer Methods in BASIC* (Von Nostrand Reinhold, 1982).
2. Norris, A. C., *Computational Chemistry* (John Wiley and Sons, 1981).

### ANALYTICA 86

*3 to 6 June 1986 at the Munich Trade Fair Centre*

The scientific programme for this international meeting is divided into symposia, posters and the 'Analytica – Forum München' (in this latter sector, exhibiting companies will present papers on developments in industrial research). Topics to be covered include:

Separation methods
- Chromatographic methods
- Electrophoretic methods

Combined methods: MS-MS, HPLC-MS, GC-MS, GC-IR, LC-MS, LC-NMR, FT-GC, GC-FTIR
Emission spectroscopy
Bioluminescence, chemiluminescence, fluorimetry
NMR, its application *in vivo*
Radiochemical procedures
Topochemical procedures
Enzymatic analysis
Cell and organ culture based analysis
Dry support reagents including stick tests
Progress in development of reference methods.

*Enquiries about the commercial exhibition and registration to Münchener Messe- und Ausstellungsgesellschaft mbH, Analytica 86, Postfach 12 10 09, D 8000 München 12, FR Germany. Information about scientific contributions from Professor Dr H. Feldman, Inst. für Physiologische Chemie der Universität, Goethestrasse 33, D 8000 München 2, FR Germany.*

### NOTES FOR AUTHORS

*Journal of Automatic Chemistry incorporating Journal of Clinical Laboratory Automation* covers all aspects of automation and mechanization in analytical, clinical and industrial environments. The *Journal* publishes original research papers; short communications on innovations, techniques and instrumentation, or current research in progress; reports on recent commercial developments; and meeting reports, book reviews and information on forthcoming events. All research papers are refereed.

### Manuscripts

Two copies of articles should be submitted. All articles should be typed in double spacing with ample margins, on one side of the paper only. The following items should be sent: (1) a title-page including a brief and informative title, avoiding the word 'new' and its synonyms; a full list of authors with their affiliations and full addresses; (2) an abstract of about 250 words; (3) the main text; (4) appendices (if any); (5) references; (6) tables, each table on a separate sheet and accompanied by a caption; (7) illustrations (diagrams, drawings and photographs) numbered in a single sequence from 1 upwards and with the author's name on the back of every illustration; captions to illustrations should be typed on a separate sheet. Papers are accepted for publication on condition that they have been submitted only to this *Journal*.

### References

References should be indicated in the text by numbers following the author's name, i.e. Skeggs [6]. In the reference section they should be arranged thus:

to a journal

Manks, D. P., *Journal of Automatic Chemistry*, **3** (1981), 119.

to a book

Malmstadt, H. V., in *Topics in Automatic Chemistry*, Ed. Stockwell, P. B. and Foreman, J. K. (Horwood, Chichester, 1978), p. 68.

### Illustrations

Original copies of diagrams and drawings should be supplied, and should be drawn to be suitable for reduction to the page or column width of the *Journal*, i.e. to 85 mm or 179 mm, with special attention to lettering size. Photographs may be sent as glossy prints or as negatives.

### Proofs and offprints

The principal or corresponding author will be sent proofs for checking and will receive 50 offprints free of charge. Additional offprints may be ordered on a form which accompanies the proofs.

*Manuscripts should be sent to either Dr P. B. Stockwell or Ms M. R. Stewart, see inside front cover.*

